# Sympathetic modulation of hindlimb muscle contractility is altered in aged rats

**DOI:** 10.1038/s41598-023-33821-9

**Published:** 2023-05-16

**Authors:** Harumi Hotta, Kaori Iimura, Nobuhiro Watanabe, Harue Suzuki, Masamitsu Sugie, Kazuhiro Shigemoto

**Affiliations:** 1 Department of Autonomic Neuroscience, Tokyo Metropolitan Institute for Geriatrics and Gerontology, 35-2 Sakae-cho, Itabashi-ku, Tokyo, 173-0015 Japan; 2 Health Promotion Management Office, Tokyo Metropolitan Institute for Geriatrics and Gerontology, Tokyo, Japan; 3 Department of Geriatric Medicine, Tokyo Metropolitan Institute for Geriatrics and Gerontology, Tokyo, Japan

**Keywords:** Neuroscience, Physiology, Ageing, Neurophysiology

## Abstract

It has recently been demonstrated that reflex excitation of muscle sympathetic nerves triggered by muscle contraction contributes to the maintenance of tetanic force (TF) in rat hindlimb muscles. We hypothesized that this feedback mechanism between the contraction of hindlimb muscles and the lumbar sympathetic nerves declines during aging. In this study, we examined the contribution of sympathetic nerves on skeletal muscle contractility in young adult (4–9 months old, n = 11) and aged (32–36 months old, n = 11) male and female rats. The tibial nerve was electrically stimulated to measure the TF of the triceps surae muscles resulting from motor nerve activation before and after cutting or stimulating (at 5–20 Hz) the lumbar sympathetic trunk (LST). The TF amplitude decreased by cutting the LST in the young and aged groups; however, the magnitude of the decrease in TF following transection of the LST in the aged rats (6.2%) was significantly (*P* = 0.02) smaller compared with that in the young rats (12.9%). The TF amplitude was increased by LST stimulation at ≥ 5 Hz in the young and ≥ 10 Hz in the aged groups. The overall TF response to LST stimulation was not significantly different between the two groups; however, an increase in muscle tonus resulting from LST stimulation, independent of motor nerve stimulation, was significantly (*P* = 0.03) greater in aged compared with young rats. The sympathetic contribution to support motor nerve-induced muscle contraction declined, whereas sympathetic-mediated muscle tonus, independent of motor nerve activity, was augmented in aged rats. These changes in sympathetic modulation of hindlimb muscle contractility may underlie the reduction of skeletal muscle strength during voluntary contraction and rigidity of motion during senescence.

## Introduction

Senescence is accompanied by progressive sarcopenia, which is a marked decline in muscle mass, strength, and motor function in the extremities^1–3^. Sarcopenia not only interferes with daily life, but also doubles the risk of death^4,5^, making it an important issue to be addressed. Muscle strength is dependent on muscle mass; however, in sarcopenia, the extent of muscle strength reduction is greater compared with that in muscle mass. The cause of this discrepancy between muscle mass and strength is unclear, but it may involve an increase in muscle stiffness^6–8^ or a decline in nerve regulation^9–14^. In addition to the motor nerves, skeletal muscles are innervated by autonomic nerves. To determine the cause of age-related sarcopenia, it is important to determine how the autonomic nervous system, in addition to the muscles themselves and the motor nervous system, influences skeletal muscle function throughout life.

Hindlimb muscle nerves contain unmyelinated sympathetic postganglionic fibers, for example, which account for 40% of all nerve fibers in the combined nerve of the lateral gastrocnemius and soleus muscle of the cat^15^. These sympathetic nerve fibers appear to be distributed not only in vascular smooth muscle, but also in skeletal muscle fibers^16^ and neuromuscular junctions^17–19^. We identified a feedback mechanism between skeletal muscle and sympathetic nerves, in which reflex excitation of the lumbar sympathetic nerves induced by contractions of the hindlimb muscles modulates their contractility^20^. Motor nerve stimulation-induced tetanic force (TF) amplitude was reduced by approximately 10% of the original force at 20 min following transection of the lumbar sympathetic trunk (LST), spinal cord, or dorsal roots. This feedback mechanism assists motor nerve function, so that a reduction in this sympathetic mechanism may lead to a reduction of muscle force in the elderly.

There is evidence for adrenergic receptors on both the skeletal muscle fiber itself and on the cholinergic motor nerve terminal^17^ and each target has the potential to alter the contractility of the muscle fiber when activated. The β-receptor-mediated increase in frequency of the miniature endplate potential of dissected hindlimb muscle resulting from sympathetic stimulation was reduced in aged mice (31 months old) compared with young mice (3–4 months old), suggesting a decline in the sympathetic regulation of cholinergic nerve terminals during aging^21^. On the contrary, α-receptor stimulation increased muscle tonus in the dissected hindlimb muscles of very old rats (36 months old), but not in those of young adult (5–7 months old) or aged rats (24 months old)^22^. This response was dependent upon extracellular Ca^2+^, but was not affected by curare; thus, it was apparently independent of the neuromuscular junction. Therefore, aging may increase α receptor function in skeletal muscle fiber itself. Nonetheless, we could not find a study that examined whether the contribution of in vivo physiological sympathetic nerve activity on muscle contractility changes during aging.

The resting activity of the sympathetic nerve increases with age as evidenced by recording muscle sympathetic nerve discharge in humans^23–25^ or adrenal sympathetic nerves in rats^26^, or by measuring plasma noradrenaline concentrations in rats^27^ and humans^28^. Although muscle sympathetic nerve tonus increases with age, sympathetic-mediated regulation of contractility may be altered. Thus, the relationship between muscle sympathetic activity and skeletal muscle function changes with aging should be carefully examined.

We hypothesized that the sympathetic contribution to TF generation by motor nerves declined, whereas sympathetic-mediated muscle tonus, independent of motor nerve activity, was augmented during aging. In this study, we determined the relationship between lumbar sympathetic nerves and muscle contractility in male and female young adult and aged rats. Parts of this study have already been presented in the form of an abstract^29^.

## Methods

### Basic preparation

Experiments were performed on 11 male and 11 female Fischer rats (young adult group 4–9 months old, aged group 32–36 months old; weight: male 310–405 g, female 181–277 g) (Table 1). The experiments were conducted in accordance with the “Guidelines for the Proper Conduct of Animal Experiments” established by the Science Council of Japan in 2006, and were approved by the Animal Experiment Committee of the Tokyo Metropolitan Institute of Gerontology. This study is reported in accordance with ARRIVE guidelines.

**Table 1:** Characteristics of the rats.

	Young adult (4–9 months)		Aged (32–36 months)	
	Male (n = 6)	Female (n = 5)	Male (n = 5)	Female (n = 6)
Body weight, g	365 (310–380)	198 (181–206)	376 (350–405)	246 (212–277)
Weight of the triceps surae muscles, g	2.1 (1.8–2.2)	1.2 (1.1–1.2)	1.2 (0.9–1.5)	0.9 (0.8–1.0)
Relative weight of the muscles, % of body weight	0.58 (0.56–0.60)	0.59 (0.57–0.64)	0.32 (0.24–0.39)*	0.36 (0.35–0.39)^#^
Basal TF amplitude, g	1.93 (0.71–2.61)	1.40 (1.32–1.94)	1.43 (0.12–4.13)	1.58 (0.33–3.55)

Values are presented as median and range.

*P < 0.0001 vs. young males, ^#^*P* < 0.0001 vs. young females by one-way factorial ANOVA and Bonferroni’s multiple comparisons test.

The methods used in this study, including basic preparations, such as artificial respiration, recording blood pressure and muscle tension of triceps surae muscles, and lumbar sympathetic nerve transection and stimulation, are essentially the same as those described previously^20^. The methods are briefly described below. Animals were anesthetized with urethane (initially 1.1 g/kg, s.c.), while respiration and body temperature were maintained physiologically. A catheter was inserted into the jugular vein for drug administration and a catheter for monitoring systemic arterial pressure was inserted into the common carotid artery. At the end of the experiment, the rats were euthanized by injecting a large dose of secobarbital, the left triceps surae muscles were removed, and wet weight was measured.

### Recording of muscle tension

After two silver wire electrodes were implanted in the intact unilateral tibial nerve, the ipsilateral hindlimb was fixed in the plantar flexed position, and the Achilles tendon was connected with a thread to a force transducer (Ultra Small-Capacity Load Cell, LVS-20GA, Kyowa Electronics Instruments, Tokyo, Japan, sensitivity 200 mN) and an amplifier (AP-610J, Nihon Kohden, Tokyo, Japan) to measure isometric force. The force transducer was placed as the angle from the longitudinal axis of the triceps surae muscle to be 90 degrees. The tibial nerve was electrically stimulated with a 0.2 ms rectangular pulse using a stimulator (SEN-340MG, Isolator, SS-203JMG, Miyuki Giken, Tokyo, Japan). The current intensity was set to twice that of the motor threshold (2 T) to excite all motor fibers without directly exciting small diameter fibers, such as sympathetic postganglionic nerve fibers and group III and IV afferent nerve fibers^30^. Group I afferent nerve fibers excited by the 2 T intensity are known not to have any influence on the sympathetic nervous system (see a review^31^). A tetanic contraction was induced by train stimulation (10 pulses at 100 Hz). Ten successive tetanic contractions at 1 Hz were repeated three times at 1 min intervals. The waveforms of these 30 contractions were averaged and treated as one set of contractions. The value of the tetanic force (TF) of the muscle contraction varied depending on the degree of muscle stretching. We intended to consider small contractions in the elderly’s daily life. Therefore, care was taken not to stretch the muscles, so we used just a small weight of 0.2 g to make the thread horizontal without sagging, and then the thread was fixed. In this setting, the force amplitude was < 5 g, allowing us to use the high sensitivity load cell, which enabled us to simultaneously examine slight changes in muscle tonus at rest.

### Transection and stimulation of the LST

The unilateral LST, on the side ipsilateral to the measured muscles, was exposed and cut near the renal artery. The peripheral end of the severed LST was placed over a bipolar stimulating electrode of platinum-iridium wire and the nerve was covered with warm paraffin oil. Stimulus trains were applied at frequencies of 5 Hz, 10 Hz, and 20 Hz at supramaximal intensity (8–10 V, 0.5 ms). LST stimulation duration was 190–200 s, starting 1 min before the onset of tibial nerve stimulation, and lasted until 0–10 s after the end of that stimulation. The basal force was averaged for 30 s before and after the onset of LST stimulation, and the difference was treated as the change in muscle tonus resulting from LST stimulation. Blood pressure was also analyzed in the same manner.

### Drugs

The α-receptor blocker phentolamine (Regitin, Novertis Pharma, Tokyo) (5 mg/kg, i.v.)^32^, the β-receptor blocker propranolol (Inderal, Taiyo Pharma, Tokyo) (1 mg/kg, i.v.)^32^, and the muscle relaxant vecuronium bromide (Fuji Pharma, Toyama) (1–2 mg/kg, i.v.)^33^ were used as described in the Results.

### Data analysis

All analog signals were digitized (Micro 1401, Cambridge Electronic Design, Cambridge, UK), displayed on a computer monitor, and analyzed online and offline using Spike2 software. Values are expressed as medians and interquartile ranges, unless otherwise stated. Normality was checked by the Kolmogorov–Smirnov test, and nonparametric tests were performed if the data points did not follow a normal distribution. Comparisons between two groups were performed by a paired *t*-test, unpaired *t*-test, or Wilcoxon signed-rank sum test (two-tailed). Comparisons between aged and young rats for the magnitude of response to LST stimuli (5–20 Hz) were analyzed by a two-way repeated measures analysis of variance (ANOVA) (Prism 9, GraphPad Software, CA, USA). Comparisons between the four groups were made by a one-way factorial ANOVA or Kruskal–Wallis test, and when significant, post-hoc comparisons were made between males and females of the same age group and between young and aged groups of the same sex. *P* values were presented after Bonferroni’s correction. Statistical significance was set at *P* < 0.05.

## Results

The values for the weight of both whole body and unilateral triceps surae muscles and the relative weight of the muscles expressed as percent body weight are shown for young males (n = 6), young females (n = 5), aged males (n = 5), and aged females (n = 6) in Table 1. Although males and females differed by nearly two-fold in body weight and muscle weight, relative triceps surae muscle weight per body weight was similar and did not differ between males and females in the young adult group, with a median of 0.58% (range: 0.56%–0.60%) in males and 0.59% (0.57%–0.64%) in females. In contrast, a significant (*P* < 0.0001) reduction of 55% and 61%, respectively, to 0.32% (0.24%–0.39%) and 0.36% (0.35%–0.39%) was observed in aged males and females compared with young males and young females, respectively (Table 1). Aged males exhibited greater individual differences in relative muscle weight compared with the other three groups.

### Effect of LST transection on TF

**Figure 1:**
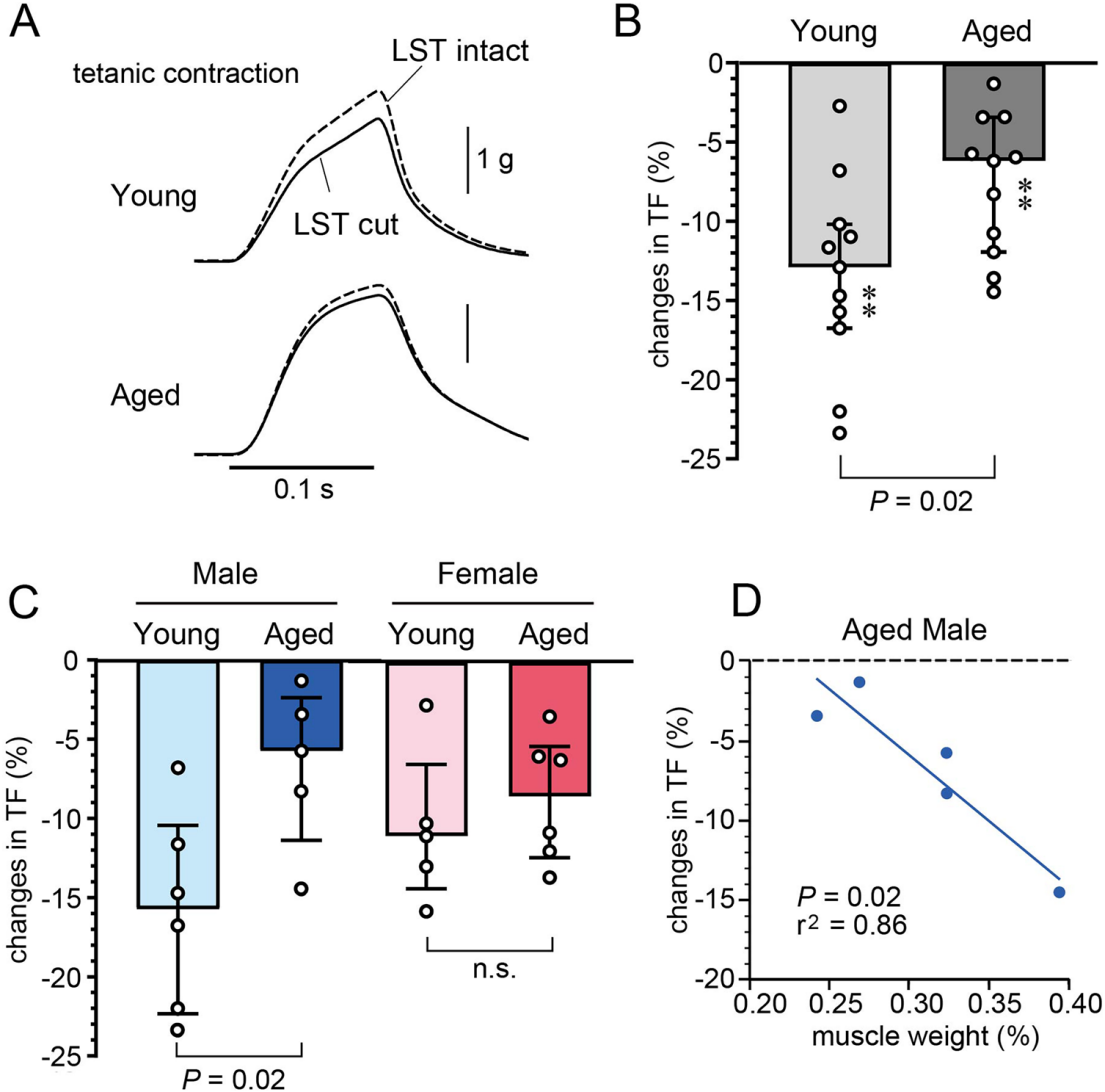
Effect of lumbar sympathetic trunk (LST) transection on the tetanic force (TF) of the triceps surae muscles. (**A**) Example responses in each young and aged rat of the TF induced by tibial nerve stimulation before (LST intact) and 20 min after LST transection (LST cut). The traces show the superimposed contraction curves, each averaged over 30 contractions before (dotted line) and after (solid line) LST transection. (**B**) Graph summarizing the changes in the amplitude of the TF in the young adult (n = 11 rats) and aged (n = 11 rats) groups, expressed as the percent change of control TF before LST transection. **P < 0.01 (paired *t*-test, vs. control TF with intact LST). (**C**) Subgroup analysis of age and sex. (**D**) Correlation between muscle weight (% of body weight) and changes in TF (% of control force) induced by the LST cut was tested by simple linear regression. Each column, vertical bar, and circle indicates the median, interquartile range, and individual data, respectively.

Tetanic contractile force was reduced after transection of the lumbar sympathetic trunk in all animals. Figure 1A shows examples of tetanic contraction of the triceps surae muscles in a young and an aged rat under control conditions with intact and transected LST. Each panel shows the superimposed contraction curves for two conditions, which were averaged over 30 contractions. The peak amplitude of the TF was slightly reduced by the LST transection.

The tetanic force amplitude in the control condition with intact LST ranged from 0.1 to 5 g in individual rats (Table 1) and did not significantly differ among groups (*P* > 0.97, by Kruskal–Wallis test). In accordance with our previous result^20^, there was a strong correlation between the original TF amplitude and magnitude of changes in TF after cutting the LST in both young and aged animals (Supplementary Fig. S1). Therefore, we compared relative changes in TF following LST cut between young and aged groups. Figure 1B summarizes the results of the percent changes in TF amplitude resulting from LST transection in 11 young adult and 11 aged rats. In the young adult and aged groups, the amplitude of TF was significantly reduced after cutting the LST compared with the control condition with intact LST (*P* < 0.01, by paired *t*-test). However, the degree of decrease was 6.2% in the aged group, which was half of that in the young adult group (12.9%). There was a significant difference between values in the young and aged groups (*P* = 0.02, unpaired *t*-test) (Fig. 1B).

Next, to examine sex differences, a subgroup analysis was performed in four groups, with each value in the young adult and aged group divided by sex. The magnitude of TF reduction in young males, aged males, young females, and aged females was 15.7%, 5.8%, 11.0%, and 8.5%, respectively (Fig. 1C) and there was a significant difference by one-way factorial ANOVA (*P* = 0.04). The post-test showed a significant difference between the young and aged male groups (*P* = 0.02), but not between the young and aged female groups (*P* = 0.9). Because the individual variation in the relative muscle weight was large in aged males, as described above, we examined the correlation between relative muscle weight and the rate of TF reduction by LST transection in the aged male group and observed a strong correlation (r^2^ = 0.86, *P* = 0.02) between these two parameters (Fig. 1D). However, the same analysis in the aged female group did not show a significant correlation (r^2^ = 0.39, *P* = 0.18).

### Effect of LST stimulation on TF

**Figure 2:**
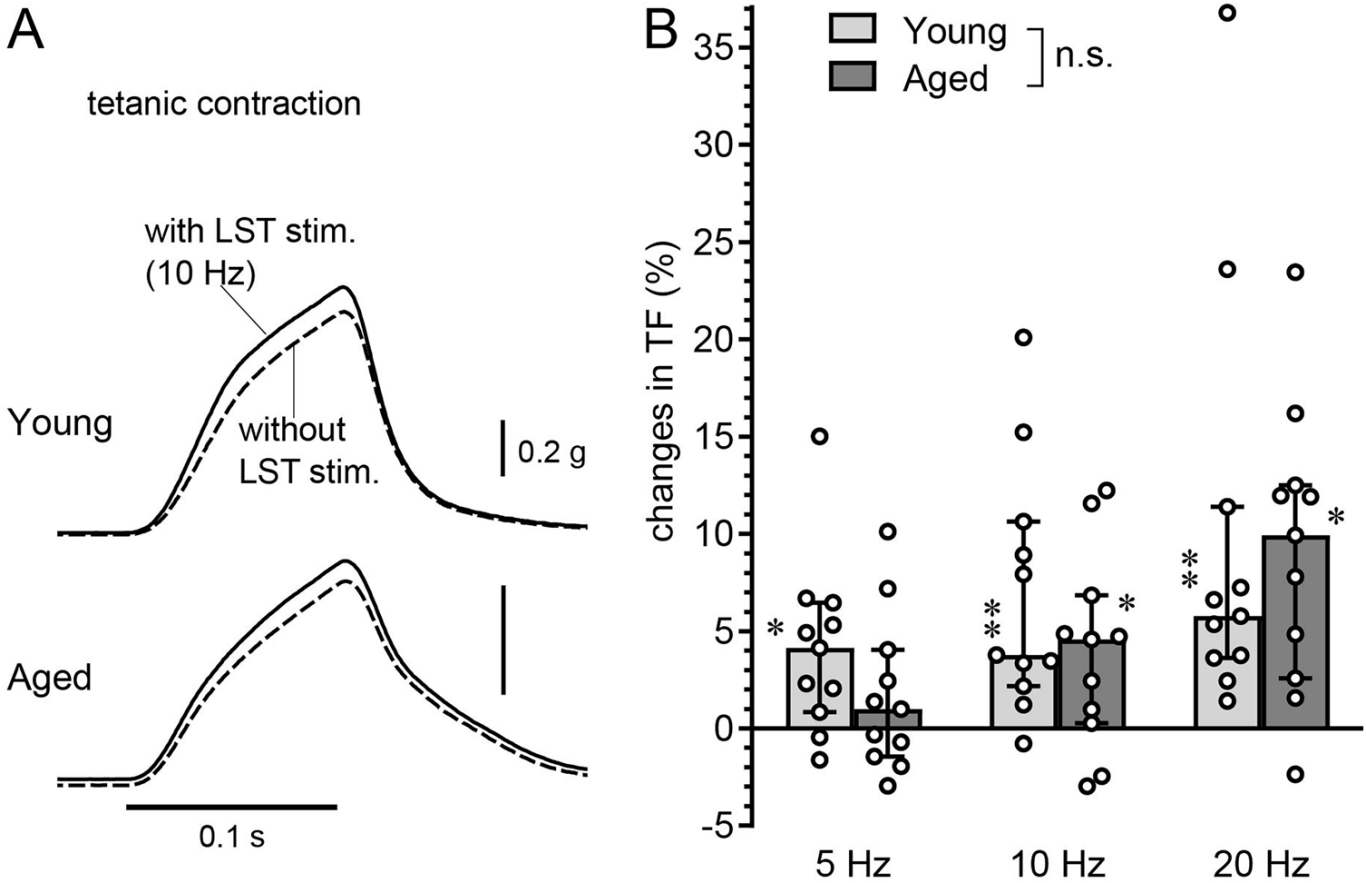
Effect of LST stimulation on the TF of the triceps surae muscles. (**A**) Example responses in each one young and aged rat of the TF before and during LST stimulation at 10 Hz. See Fig. 1A for further details. (**B**) Graph summarizing (n = 11 rats for each group) the changes in the amplitude of the TF expressed as the percent change of the control TF without LST stimulation. X axis: frequency of LST stimulation. Y axis: changes in the peak amplitude of the TF during LST stimulation, expressed as a percentage of the control TF just before LST stimulation. *P < 0.05, **P < 0.01 (paired *t*-test, compared with the control TF without LST stimulation).

Stimulation of the lumbar sympathetic trunk could increase tetanic contractile force. Figure 2A shows TF recordings with and without stimulation of the peripheral end of the transected LST at a frequency of 10 Hz in a young and an aged rat. In both groups of rats, TF increased slightly with LST stimulation (solid line) compared to that without (broken line). The effect of LST stimulation was dependent upon the frequency of stimulation (Fig. 2B). In young adult rats, TF was significantly increased at 5 Hz to 20 Hz compared to the pre-stimulus control TF (*P* < 0.05, by paired t-test), which is consistent with a previous report^20^. In contrast, in aged rats, there were no significant changes at a frequency of 5 Hz (*P* = 0.19); however, a significant increase was observed at frequencies of 10 Hz and 20 Hz (*P* < 0.05). The increase in TF in response to LST stimulation at 5 Hz, 10 Hz, and 20 Hz was 4.1%, 3.8%, and 5.8% in the young group and 0.9%, 4.6%, and 9.9% in the aged group, respectively. A two-way ANOVA indicated that, although there was a significant difference in the primary effect of stimulus frequency, there was no significant difference in the effect of aging or interaction. The median of the response to 5 Hz LST stimulation was smaller in aged rats compared with that in young rats; however, the individual variation was large, and the difference was not statistically significant.

Finally, a one-way factorial ANOVA of TF responses to 5 Hz, 10 Hz, and 20 Hz LST stimuli, respectively, in the four groups (young males, aged males, young females, and aged females) revealed no significant differences at any stimulus frequency (Supplementary Figure S2A).

### Effect of LST stimulation on muscle tonus

**Figure 3:**
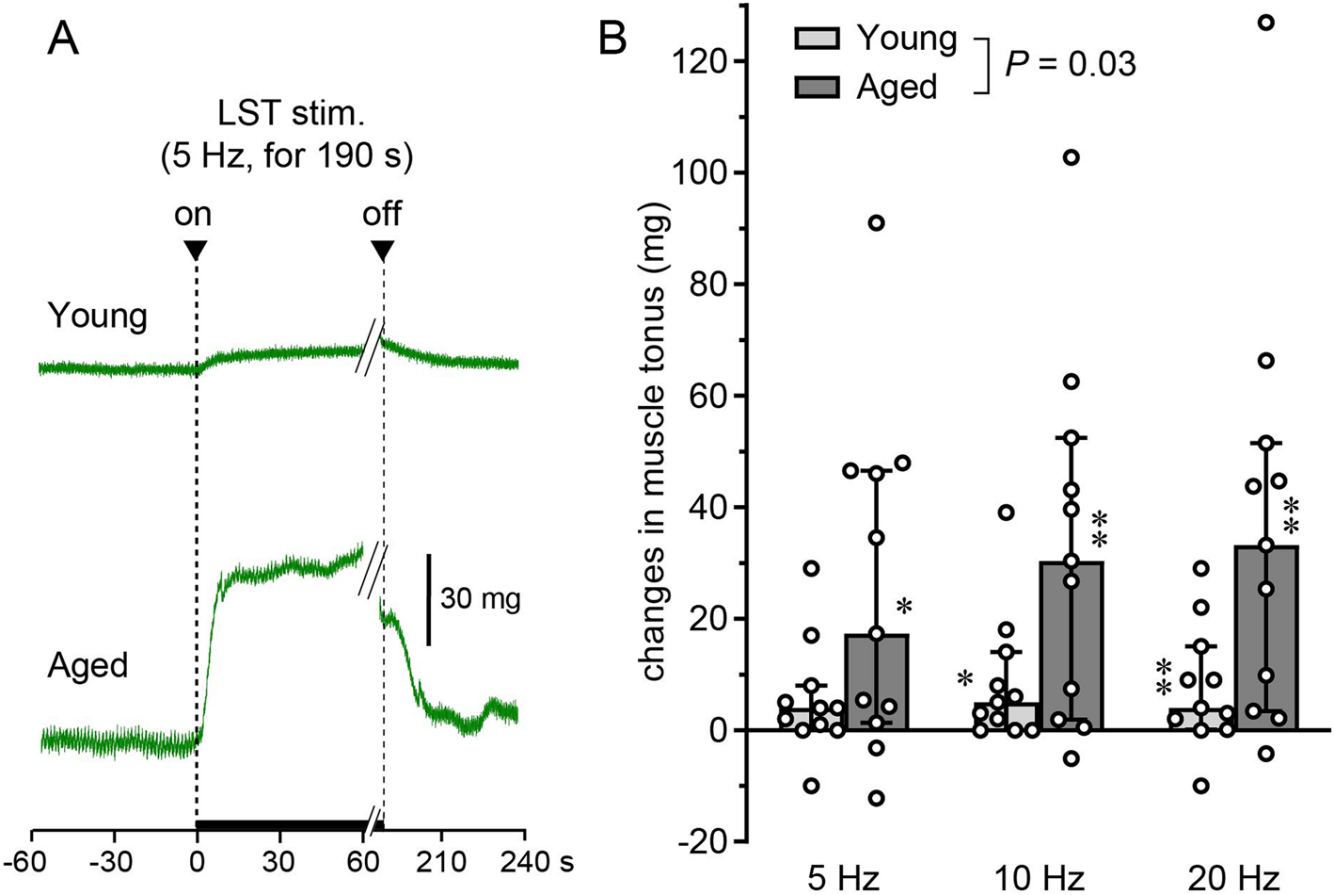
Effect of LST stimulation on the tonus of the triceps surae muscles. (**A**) Example responses in one young and one aged rat of the response of muscle tonus induced by LST stimulation at 5 Hz. Among a 190 s period of LST stimulation, a 129 s period including tibial nerve stimulation was omitted. (**B**) Graph summarizing (n = 11 rats for each group) the changes in muscle tonus. X axis: frequency of LST stimulation. Y axis: changes in muscle tonus during LST stimulation, expressed as a delta value relative to the control level (or tonus) just before LST stimulation. *P < 0.05, **P < 0.01 (Wilcoxon signed-rank sum test, compared with the pre-stimulus control).

Lumbar sympathetic trunk stimulation increased resting muscle tonus. LST stimulation was initiated 1 min before the onset of tibial nerve stimulation. Therefore, the effect of LST stimulation alone could be observed, independent of motor nerve excitation. Figure 3A shows that LST stimulation alone increased muscle tonus in young and aged rats, but the magnitude of the response was much larger in aged than in young rats. The tension started to increase within 2 s after the onset of LST stimulation and reached nearly its maximum approximately 10 s later. The tension remained increased during stimulation for 190 s, and then returned to the original level within 20 s after the cessation of the stimulation. This increase in tension of more than 20 mg was observed in 7 of 11 rats in the aged group and in only 2 of 11 rats in the young adult group. In some cases (one young and one aged rat), a small decrease in tension, by approximately 10 mg, was observed.

The effect of LST stimulation on increasing muscle tonus was dependent upon the frequency of stimulation. In young rats, stimulation at 10 Hz and 20 Hz, but not 5 Hz, produced significant responses (*P* < 0.05). In contrast, in aged rats, stimulation at frequencies of 5–20 Hz was effective at producing significant increases in muscle tonus (*P* < 0.05). The increase in muscle tonus in response to LST stimulation in the aged group was 17 mg, 30 mg, and 33 mg at 5 Hz, 10 Hz, and 20 Hz, respectively, and was 4- to 8-times greater compared with that of the young group, which was 4 mg, 5 mg, and 4 mg, respectively (Fig. 3B). Two-way ANOVA revealed a significant difference in the main effect of aging (*P* = 0.03). Changes in muscle tonus in response to LST stimulation at frequencies of 5 Hz, 10 Hz, and 20 Hz showed similar trends in males and females of the same age group (Supplementary Fig. S2B).

LST stimulation at 5–20 Hz resulted in an increase in blood pressure in all rats, regardless of age or sex. The magnitude of the blood pressure increase at 5 Hz, 10 Hz, and 20 Hz stimulation was 8 mmHg, 13 mmHg, and 14 mmHg in the aged group, respectively, which was significantly lower by approximately 30–40% compared with 11 mmHg, 17 mmHg, and 24 mmHg in the young group. There were significant differences between the young and aged groups by two-way ANOVA (*P* < 0.01). These results were similar in both male and female rats (Supplementary Fig. S3).

### Involvement of adrenergic receptors on LST stimulation-induced modulation of TF and muscle tonus

#### Tetanic force

**Figure 4:**
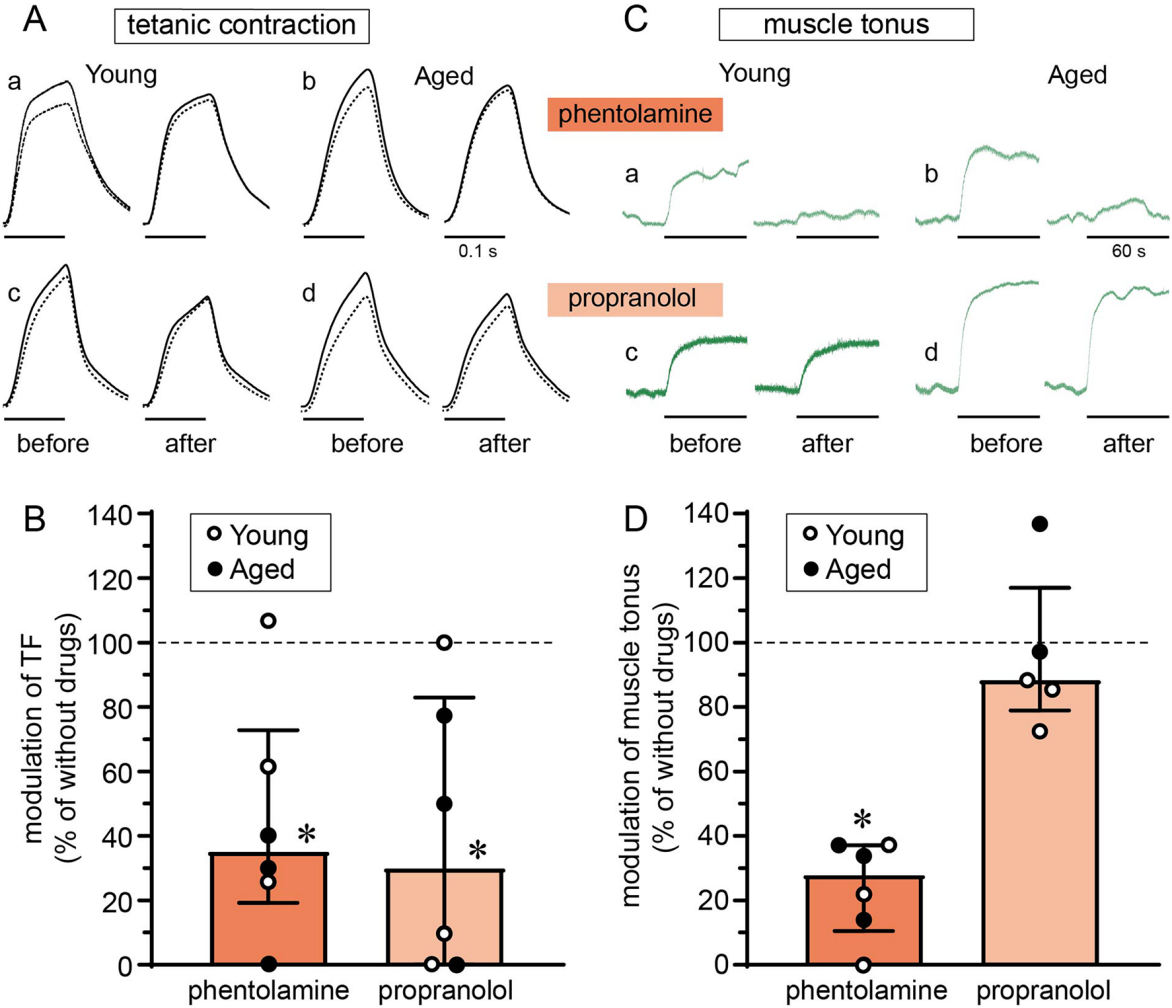
Effect of adrenergic blockers on the LST stimulation-induced changes in TF (**A,B**) and muscle tonus (**C,D**) of the triceps surae muscles. (**A,C**) Example responses in each young (**a,c**) and aged (**b,d**) rat of the response induced by LST stimulation at 10 or 20 Hz before and after phentolamine (**a,b**) or propranolol (**c,d**) administration. (**B,D**) Graph summary. n = 5–6 rats, including young (open circles) and aged (closed circles) rats. The extent of modulation was expressed as the percent of the control value before administration of phentolamine or propranolol. *P < 0.05 (paired *t*-test, compared with the control response before injection).

Alpha or beta adrenergic receptor blockade could each reduce the impact of LST stimulation on tetanic contractile force. The effect of adrenergic receptor blockade was examined in cases in which LST stimulation at 10 Hz or 20 Hz increased TF by more than 4%, irrespective of age or sex. Figure 4Aa, b shows typical examples of the effect of the α adrenoceptor blocker phentolamine in a young (a) and an aged (b) rat. After phentolamine administration, the magnitude of TF without LST stimulation (dashed line) did not change compared with that before phentolamine administration; however, TF augmentation resulting from LST stimulation (difference between dashed and solid lines) observed before phentolamine injection was reduced after phentolamine in both rats. When summarized for six cases, the LST stimulation-induced modulation of TF was significantly (*P* < 0.05) attenuated to approximately 35% of the control response before phentolamine administration (Fig. 4B, dark column), without a significant change in basal TF values. Next, the effect of the β receptor blocker propranolol was determined in a different set of rats, as shown in typical examples in a young (Fig. 4Ac) and an aged (Fig. 4Ad) rat. Propranolol also significantly reduced the LST stimulation-induced modulation of TF to 30% of the control response (n = 6, *P* < 0.05) (Fig. 4B, light-colored column). However, after propranolol administration, the basal TF values in the absence of LST stimulation were slightly but significantly (*P* < 0.01) reduced compared with those before propranolol administration (91% of the original). There were individual differences in the effect of each α and β blocker, as indicated by the dots in the graphs. When a majority of the response remained with either blocker, a second blocker was administered. Then, the LST-induced increase in TF completely disappeared (4 animals, 2 with phentolamine followed by propranolol and 2 with phentolamine followed by propranolol). These results indicate that both α- and β-receptors are involved in the LST stimulation-induced potentiation of the TF. These results were somewhat similar to a previous report in rabbits, in which the potentiation of jaw muscle twitch contraction resulting from cervical sympathetic trunk stimulation was minimally affected by propranolol, but was attenuated with a subsequent injection with phentolamine^34^. Two-way ANOVA revealed that there was no significant main effect of age or drug type, and no significant interaction (n = 3 for each group, *P* > 0.3).

#### Muscle tonus

Alpha adrenergic receptor blockade, but not beta receptor blockade, eliminated the impact of LST stimulation on muscle tonus. The effect of adrenergic receptor blockers was examined in cases in which LST stimulation (10 Hz or 20 Hz) increased muscle tonus. Figure 4Ca, b shows typical examples of the effect of phentolamine in a young (a) and an aged (b) rat. Following phentolamine administration, the increased response of the muscle tonus resulting from LST stimulation was nearly abolished in both young and aged rats. To summarize the six cases, the magnitude of the LST stimulation-induced muscle tonus increase was significantly (*P* < 0.05) ameliorated to approximately 28% of the control response before phentolamine (Fig. 4D, dark column). In contrast, administration of propranolol (see typical examples in Fig. 4Cc, d) had no significant effect on the increased muscle tonus response (Fig. 4D, light-colored column). The LST stimulation-induced increase in muscle tonus was minimally affected by administration of the muscle relaxant vecuronium, which was evaluated in three rats. These results indicate that LST stimulation induced an increase in muscle tonus, independent of motor nerve activation, via α-receptors, but not β-receptors. There was no significant difference in the modulation of muscle tonus by phentolamine between the young and aged groups (n = 3 for each group, *P* > 0.5).

## Discussion

The major finding of this study was that the feedback mechanism between the contraction of hindlimb muscles and the lumbar sympathetic nerves declined in aged animals. Based on the results from transecting the LST, cervical spinal cord, or lumbar dorsal roots, and also from recordings of reflex potentials evoked in lumbar sympathetic nerves, we demonstrated that information from contracting muscles triggers spinal and supraspinal somato-lumbar sympathetic reflexes that help maintain contractility^20^. In the present study, the sympathetic contribution of the TF amplitude, as determined by LST transection, in aged animals was reduced to half of that in young animals.

The age-related reduction of the sympathetic contribution on TF, determined by LST transection, was significant in males, but not in females. Individual differences in triceps surae muscle weight per body weight were greater in aged males (Table 1) and smaller muscle mass was correlated with a smaller sympathetic contribution to TF (see Fig. 1D). One study found that the removal of sympathetic postganglionic neurons caused muscle atrophy after 7 days^19^; thus, it is possible that a smaller sympathetic contribution to TF may accelerate the age-related loss of muscle mass. In humans^35^ and animals (mice)^36^, the age-related decline in physical activity is reported to be more profound in males compared with females. It would be interesting to examine whether an increase in muscle mass of aged males by increasing daily exercise is associated with an increase in the sympathetic contribution to TF.

There are two possible mechanisms for the age-related decline of the sympathetic contribution on TF: (1) reduced sympathetic nerve response to muscle contraction, and (2) reduced sympathetic action, including the function of the adrenergic receptors, most likely on neuromuscular junctions. A previous study found that with respect to (1), the response of an increase in hindlimb muscle sympathetic nerve activity during 20-s static or one-minute rhythmic handgrip exercise did not differ between young (20th) and old (60th) subjects^37,38^, indicating that the sympathetic response to normal muscle contraction is well maintained in the elderly. However, older subjects had smaller muscle sympathetic nerve responses to rhythmic ischemic exercise than younger subjects^38^. The previous results regarding (2) differed among the reports. Aged mice exhibited reduced numbers of β-adrenergic receptors in hindlimb muscles and nerve fibers^21,39^ and attenuated sympathetic effects on the neuromuscular junction^21^, whereas aged rats showed no reduction in skeletal muscle β-adrenergic receptor density or muscle response to chronic β-agonist administration^27,40^.

In the present study, regarding (2), the effects of sympathetic efferent stimulation on TF were examined. There was no significant age-related difference in the potentiation of the TF amplitude resulting from LST stimulation at 5–20 Hz. However, the frequency threshold required to produce a significant increase in TF was higher in aged compared with young animals (5 Hz in young and 10 Hz in aged). Resting sympathetic nerve activity in rats suggested an increase with age^27^. In the case of adrenal sympathetic nerves, the resting activity of a single sympathetic unit averaged 1.5 Hz in 100–300-day-old young adult rats, whereas it increased up to 3 Hz in 800–900-day-old aged rats^26^. This increase in sympathetic tonus may downregulate adrenergic receptors responsible for TF modulation, particularly the response to small amounts of noradrenaline (e.g., released by 5 Hz LST stimulation or released reflexively by muscle contraction). These changes may explain the increase in the frequency threshold of LST stimulation and the decrease in TF modulation by LST transection in aged rats. Resting sympathetic activity is approximately 1–3 Hz, which is out of range from the current stimulation study (5–20 Hz). We have previously shown in young rats that LST stimulation at 1 Hz had little effect on TF, and that activity of the lumbar sympathetic nerve reflexively increased during the tetanic contractions of the hindlimb muscles^20^. The increased single unitary activity of the LST can reach to 5–20 Hz during somatosensory stimulation^41^.

**Figure 5:**
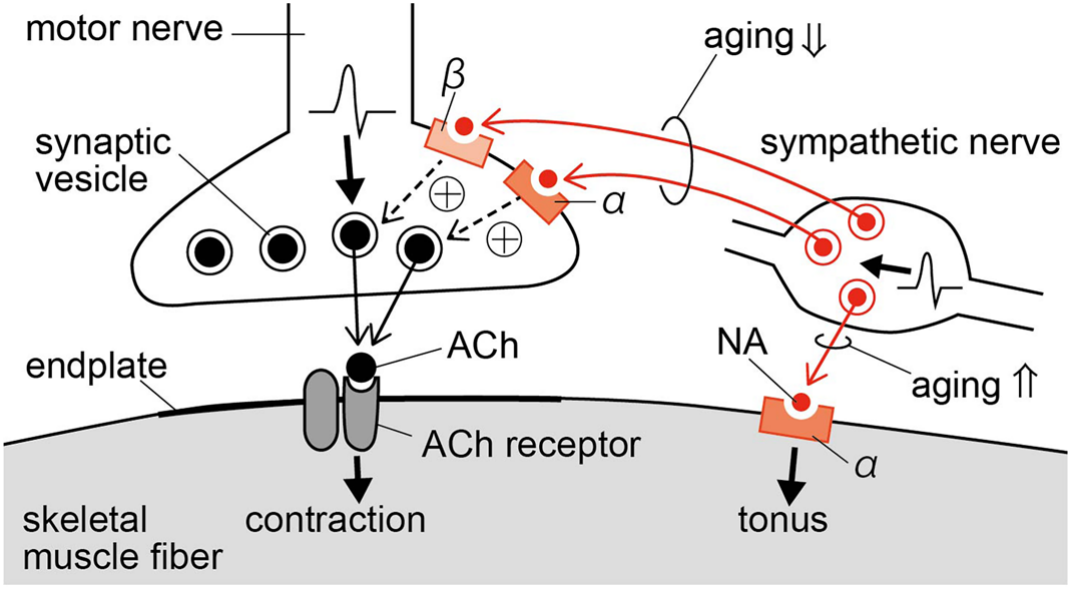
Schematic diagram showing the proposed age-related changes in the sympathetic modulation of TF (left) and muscle tonus (right).

In the present study, we showed an increase in muscle tonus resulting from LST stimulation alone, without motor nerve stimulation, in a majority of aged rats (Fig. 5, right). Carlsen and Walsh (1987)^22^ showed that pharmacological stimulation of α-adrenoceptors of the dissected flexor digitorum brevis muscle produced a neuromuscular junction-independent increase in muscle tonus in 36-month-old female rats. In the present study, we showed for the first time that an α-adrenoceptor-mediated increase in muscle tonus was induced by sympathetic nerve stimulation in aged animals.

The increased LST stimulation-induced blood pressure response was attenuated by 30%–40% in the aged group. Among the LST stimulation-induced responses in aged animals compared with young adult animals, potentiation of TF was unchanged, increase in muscle tonus was augmented, and vasoconstriction was attenuated. Although α-receptors are involved in all of these responses, the aging of each adrenergic system is different from one another. β-receptors are also involved in TF potentiation, but not in other responses. The distribution of sympathetic nerve endings and/or the distribution of adrenergic receptors in blood vessels, neuromuscular junctions, and skeletal muscle fiber membranes may change with aging. This may be related to the fact that the numbers of muscle fibers with denervated neuromuscular junctions increase with age^42,43^.

The age-related decline in the feedback system between skeletal muscle and muscle sympathetic nerves may be a factor in accelerated sarcopenia in the elderly. The age-related increase in muscle tonus with sympathetic excitation alone may be related to an increase in rigidity and deep pain often occurring in the elderly. Because the reduction in feedback mechanisms between contracting muscles and sympathetic nerves is correlated with the degree of muscle atrophy, preventing or restoring muscle atrophy through exercise may help to restore muscle-sympathetic feedback function. While TF potentiation by sympathetic nerve excitation is thought to assist motor neuron function, muscle tonus caused by sympathetic nerve excitation alone may disturb motor neuron function, because it occurs independently of motor neuron excitation. Both a decrease in sympathetic TF modulation and an increase in the sympathetic generation of muscle tonus may be related to a decline in motor function associated with aging.

In the present study, we confirmed that LST cutting or stimulation modifies the amplitude of TF in the hindlimb muscle and showed that this effect occurs in females and males, and is elicited through α and β adrenergic receptors. This positive feedback mechanism between sympathetic nerves and muscle contraction was diminished in aged hindlimb muscles (Fig. 5, left). Conversely, an α-adrenoceptor-mediated increase in muscle tonus, independent of neuromuscular end plates, is frequently observed during sympathetic stimulation in aged hindlimb muscles (Fig. 5, right). Our findings may help to link movement disorders with autonomic dysfunction in the elderly and in disease.

## Supplementary Information

Supplementary Figures.

## Data Availability

Data are contained with the articles and supplementary material.
