# Supplementary material for: Sympathetic modulation of hindlimb muscle contractility is altered in aged rats
Source: Sci Rep. 2023 May 16;13:7504. doi: 10.1038/s41598-023-33821-9 (PMC10188559; doi:10.1038/s41598-023-33821-9)
Supplement: Supplementary file 1 — Supplementary Figures. [file 41598_2023_33821_MOESM1_ESM.pdf]

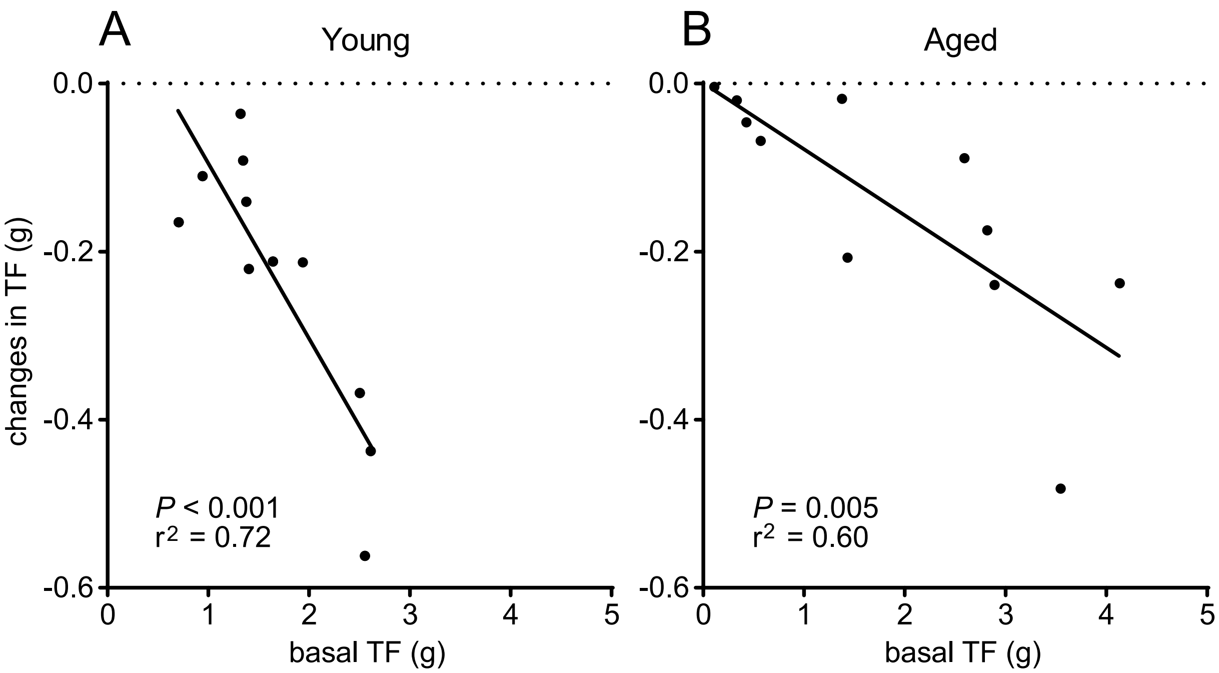

Fig. S1

Correlation between basal TF value and delta changes in TF following transection of the LST in young (A) and aged (B) rats.

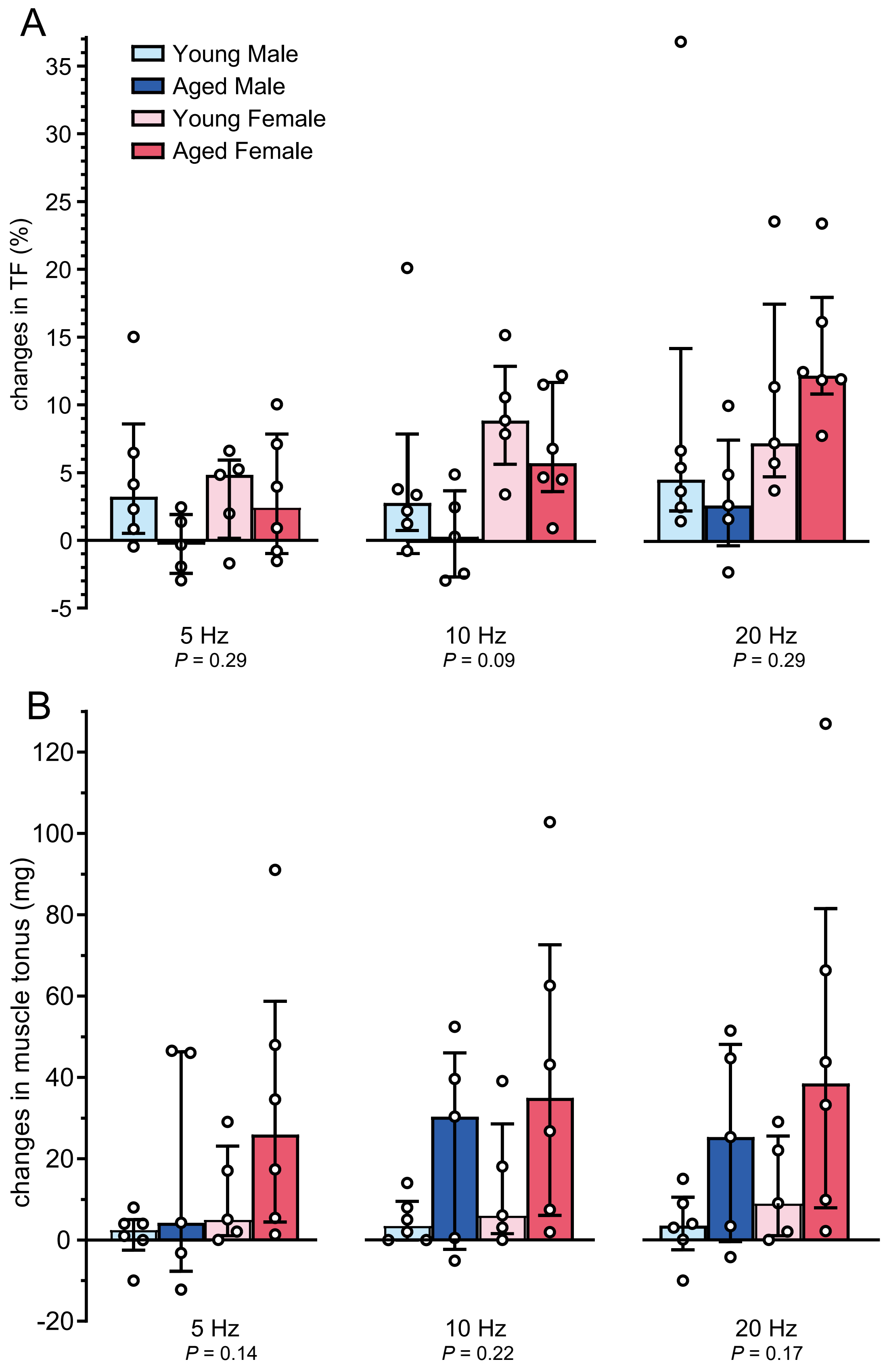

Fig. S2

Graph summarizing the changes in the amplitude of the TF (A) and muscle tonus (B) in young males, aged males, young females, and aged females.

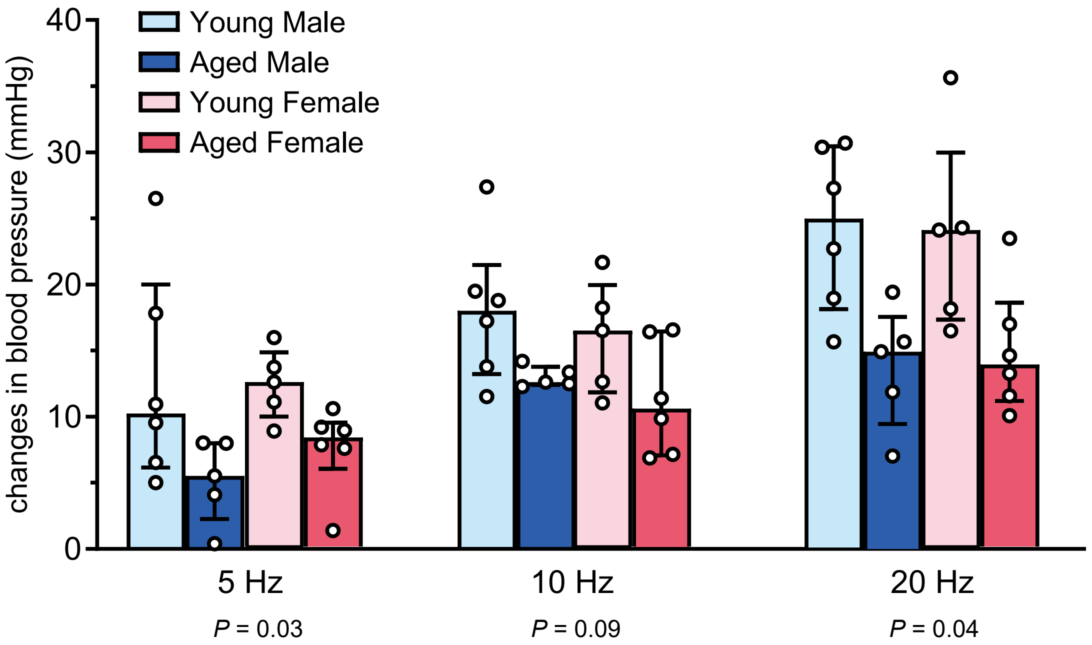

Fig. S3

Graph summarizing the changes in the blood pressure in young males, aged males, young females, and aged females.
